# Using Figurate Numbers in Elementary Number Theory – Discussing a ‘Useful’ Heuristic From the Perspectives of Semiotics and Cognitive Psychology

**DOI:** 10.3389/fpsyg.2020.01180

**Published:** 2020-06-17

**Authors:** Leander Kempen, Rolf Biehler

**Affiliations:** ^1^Department of Mathematics, University of Rostock, Rostock, Germany; ^2^Department of Mathematics, University of Paderborn, Paderborn, Germany

**Keywords:** figurate numbers, mathematical proof, proof that explain, diagrammatic reasoning, generic proof

## Abstract

The use of figurate numbers (e.g., in the context of elementary number theory) can be considered a heuristic in the field of problem solving or proving. In this paper, we want to discuss this heuristic from the perspectives of the semiotic theory of Peirce (“diagrammatic reasoning” and “collateral knowledge”) and cognitive psychology (“schema theory” and “Gestalt psychology”). We will make use of several results taken from our research to illustrate first-year students’ problems when dealing with figurate numbers in the context of proving. The considerations taken from both theoretical perspectives will help to partly explain such phenomena. It will be shown that the use of figurate numbers must not be considered to be any kind of help for learners or some way of ‘easy’ mathematics. Working in this representational system has to be learned and practiced as another kind of knowledge is necessary for working with figurate numbers. The named findings also touch upon the concept of ‘proofs that explain.’ Finally, we will highlight some implications for teaching and point to a number of demands for future research.

## Introduction

There are different ways of communicating facts and ideas in mathematics. Besides the mathematical symbolic language, geometric representations can also be used: in figurate numbers, “numbers are classified according to their geometric representation as sets of dots” (Weaver, 1974, p. 661). These figurate numbers have a long tradition in mathematics history: even the ancient Greeks and the Chinese, for example, used the geometric order of points to perform mathematics (see Chemla, 2012). As Steinweg (2002, p. 131; our translation) puts it: “Figurate numbers can be considered as a cultural heritage of mathematics.” Even today, these kinds of representations can be found in mathematical journals (e.g., Gallant, 1983; Wakin, 1984) as well as in school mathematics (e.g., Norman, 1991; Conway and Guy, 1996). While figurate numbers can constitute a unique playground for conjecturing and proving, its use can also be helpful in the context of problem solving. However, besides the useful advantages that are linked with the use of geometric representations, some research results point to possible obstacles.

In this article, we want to discuss the use of figurate numbers in mathematics from different perspectives. In this sense, each section will have a different focus on this topic. (This is why the structure of this paper is different from ‘normal’ papers with an empirical focus). The paper aims at getting deeper into the phenomenon of using figurate numbers in mathematics and in learning mathematics. This ‘deeper’ is concerned with two foci: (1) Why is it possible to do mathematics by making use of figurate numbers? (2) What problems are associated with the use of figurate numbers in mathematics for learners?

First, we will summarize the use of such representations in mathematics, especially in the field of problem solving and proving. In this context, we also want to highlight some useful benefits from the educational point of view. In a second step, we will discuss the use of figurate numbers from two theoretical perspectives. The semiotic perspective of “diagrammatic reasoning” of Peirce opens the view for the meaning of the “diagrams” used and the importance of the rules for dealing with them and reading and understanding corresponding calculations. The field of cognitive psychology will help to elaborate on the concepts of learning and understanding and hint to possible obstacles when doing mathematics in this context. In a third step, we will summarize different findings from our research to illustrate and underline the considerations taken from both theoretical perspectives. Finally, some implications for research and teaching are highlighted.

## Figurate Numbers in Mathematics and Mathematics Education

**FIGURE 1:**
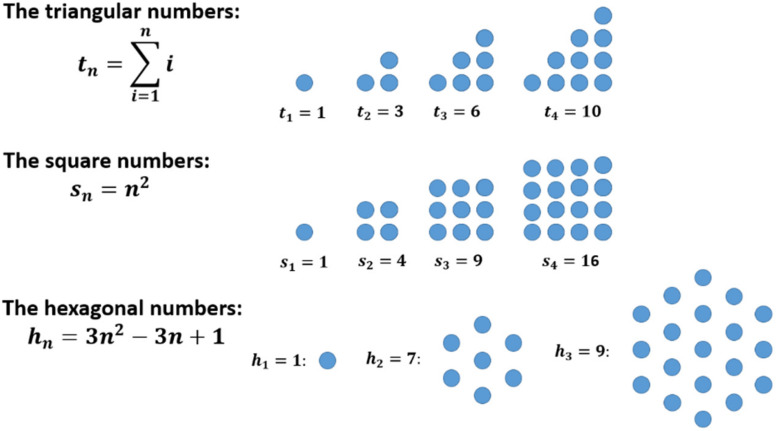
Some examples of figurate numbers (and their relationship to sequences): triangular numbers, square numbers, and hexagonal numbers.

Many sequences in mathematics can be illustrated by using a special kind of geometric representation as sets of dots and the other way round (examples are shown in Figure 1).

**FIGURE 2:**
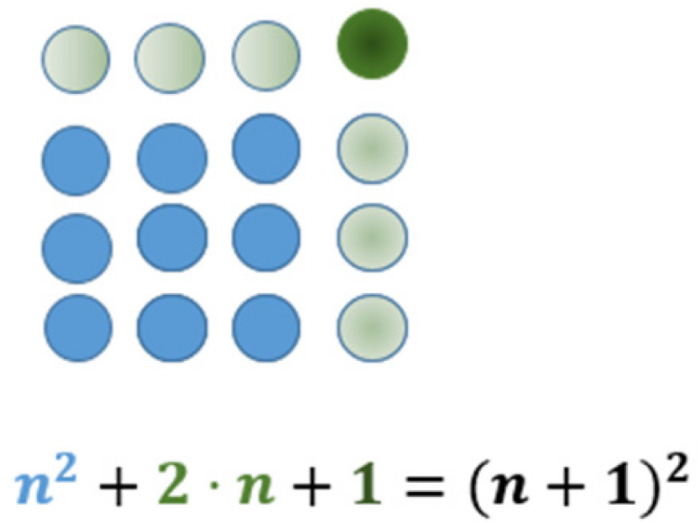
The transition from one square number to the next represented by figurate numbers.

In addition to the arithmetic properties, the geometric shapes may lead to special kinds of insights. In Figure 2, for example, the transition from a square to the next is done by adding two sides and one dot in the corner. This ‘is why’ the difference of two consecutive square numbers is always an odd number. Such types of insights that correspond to understanding have a special quality that can hardly be explained by purely behaviorist descriptions (Köhler, 1959, p. 731). We will return to this fact later in the context of cognitive psychology (see section “Some Insights From Cognitive Psychology”).

It becomes obvious, that figurate numbers can be useful for both clarifying and illuminating mathematical issues. Accordingly, they can be used in a variety of ways in mathematics and mathematics education. In the following sections, we will elaborate on three different aspects: Figurate numbers in the context of problem solving, in the context of mathematical proof and for educational purposes.

### Geometric Representations in the Context of Problem Solving

Being confronted with a ‘problem’ in mathematics, one might follow different heuristic strategies, like having a look at examples and special cases or trying to follow a forward/backward strategy. Another heuristic is using a change of representation [compare the idea of “deciding on a notation” or “change of representation to see the problem from a fresh perspective” in Mason et al. (1982) and the heuristic of variation, variation of representation, described in Schwarz (2018), p. 3 ff].

We consider the following example: “Which natural numbers can be written as a sum of consecutive natural numbers?”.

Having a look at some concrete examples, one might have different conjectures:

3=2+1;     5=2+3;     7=3+4⁢…

Conjecture: All odd numbers can be written as corresponding sums.

1+2+3=6;     2+3+4=9;     4+5+6=15;

5+6+7=18⁢…

Conjecture: The sum of three consecutive natural numbers is always divisible by three. Accordingly, numbers from the three times table can be written as consecutive sums. (It is a hypothesis to be proven that this is true for all multiples of 3).

This gives a partial answer to the initial question: all numbers from the three times table can be written as sums of consecutive numbers.

This idea can be transmitted to the sums of four consecutive numbers:

1+2+3+4=10;  2+3+4+5=14;  4+5+6+7=22;

5+6+7+8=26⁢…

In this case, the sums of four consecutive numbers are not divisible by four. However, one realizes that numbers like 10 + *n*⋅4 (*n* ∈ *ℕ*_0_) can be written as consecutive sums.

What about the sum of five consecutive numbers?

1+2+3+4+5=15;     2+3+4+5+6=20;

3+4+5+6+7=25

Conjecture: The sum of five consecutive natural numbers is always divisible by five. Accordingly, numbers from the five times table starting with 15 can be written as consecutive sums. (It is a hypothesis to be proven that this is true for all multiples of 5 greater or equal to 15).

**FIGURE 3:**
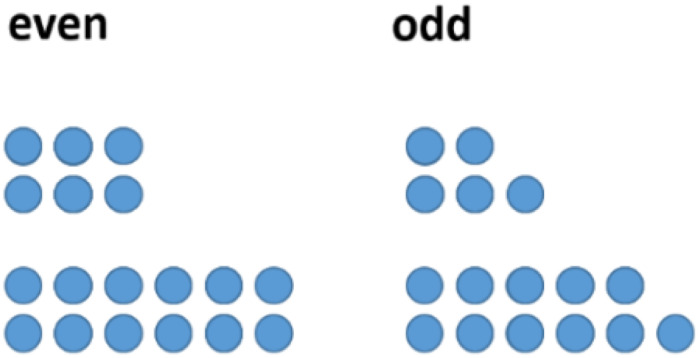
Even and odd numbers represented by figurate numbers.

**FIGURE 4:**
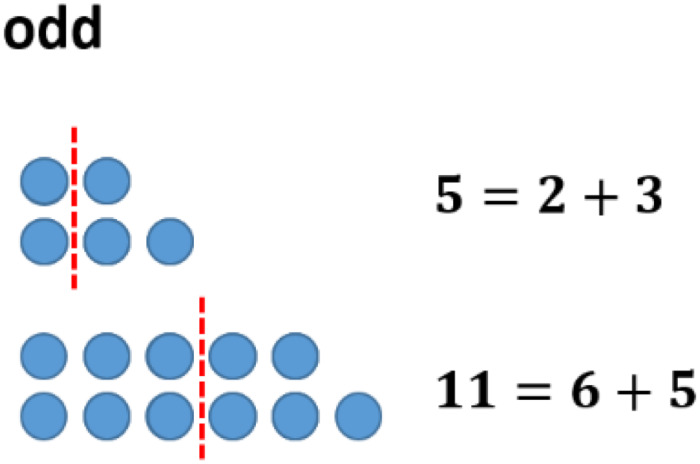
Odd numbers divided into two consecutive natural numbers.

One might follow this investigation by having a look at concrete examples. However, a change of representation can be helpful in this case. In the field of figurate numbers, even and odd numbers can be represented by two rows of dots with equal long rows (“even”) or with the difference of one dot (“odd”) (see Figure 3). Having a closer look at this structure of odd numbers, one easily divides the figure representing the odd number ‘in the middle,’ obtaining two consecutive natural numbers (see Figure 4).

**FIGURE 5:**
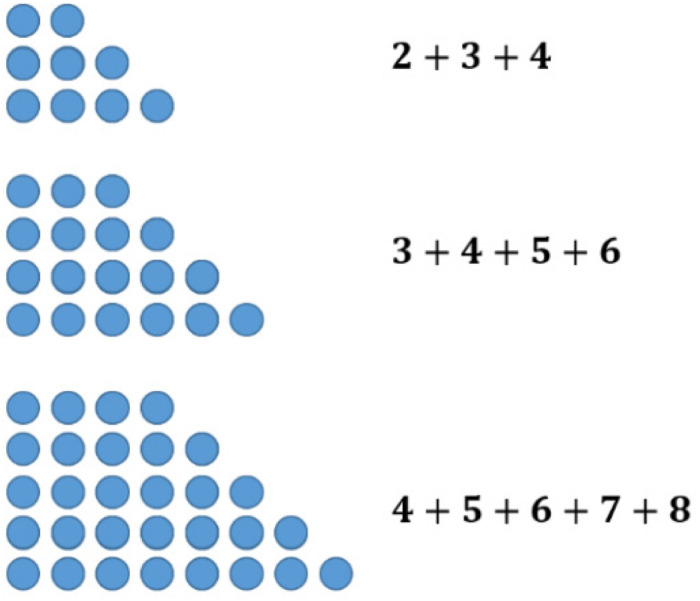
The sums of 3, 4, and 5 consecutive numbers represented by figurate numbers.

**FIGURE 6:**
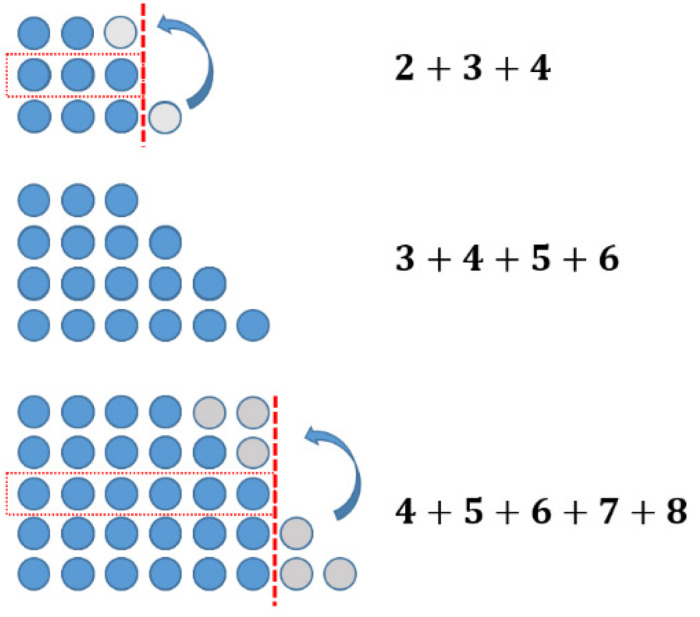
The transformation of odd numbers for obtaining equal long rows of dots.

The sums of consecutive numbers can be represented by ‘stairs’ of dots (see Figure 5). Following this idea, the sum of three consecutive numbers always has three steps, the sum of four has four, and so on. The phenomenon explaining the assumptions above is the following: having an odd number of stairs, one always has a line in the middle. Accordingly, the dots overhanging on one side can be transformed to the other side obtaining equal long rows. This transformation does not work with an equal number of stairs (see Figure 6).

Summarizing our ‘problem’: all odd numbers and all numbers that are divisible by an odd number can be written as sums of consecutive numbers. The numbers left are the powers of two (1, 2, 4, 8, 16, …). And indeed, as one can show – these numbers cannot be written as respective sums.

As we have seen, the use of figurate numbers can help investigate a problem (here: in elementary arithmetic) and may even lead to a solution. More than this, the usage of figurate numbers above also answers the question, why the assumptions are true in every case. This fact opens the view for using figurate numbers in the context of mathematical proving, too.

### Figurate Numbers and Mathematical Proof

**FIGURE 7:**
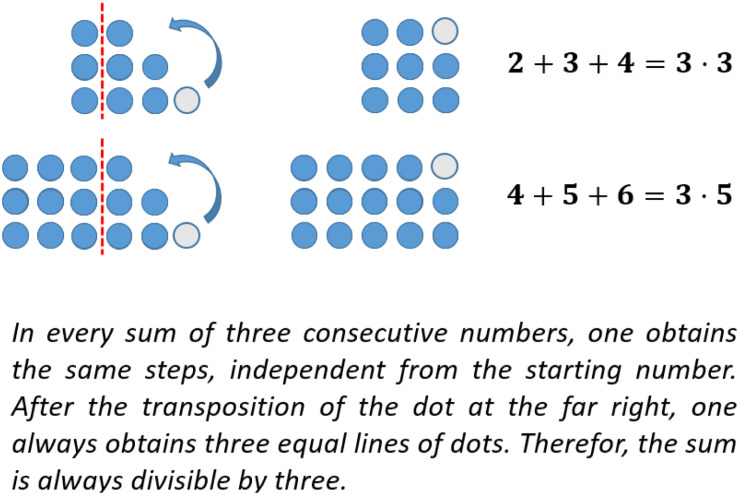
Generic proof with figurate numbers (Figure similar to Kempen and Biehler, 2019a, p. 735).

In the investigation above, an argument was found to explain, why the sum of three consecutive natural numbers is always divisible by three (see Figure 6). This idea can also be used to prove the corresponding claim. However, in the context of concrete examples, the question of generality arises. One characteristic of mathematical proof is the issue of generality. The given argument concerning the transformation of one dot to the former shortest row can be used in every possible case! This is due to the shape of stairs on the right-hand side when representing the sum of any three consecutive natural numbers by figurate numbers. This kind of proof, giving some concrete examples to illustrate an overall idea and explicating its generality is called “generic proof” (e.g., Dreyfus et al., 2012, p. 200 f.). A complete generic proof is shown in Figure 7.

In comparison, one feature of the mathematical symbolic language is the possibility to express generality, e.g., by using algebraic variables. A corresponding proof with algebraic variables might be:

For⁢all⁢n∈ℕ:n+(n+1)+(n+2)=n+n+n+1+2

=3⁢n+3=3⋅(n+1)

Since (*n* + 1) ∈ *ℕ* the sum is divisible by three.

**FIGURE 8:**
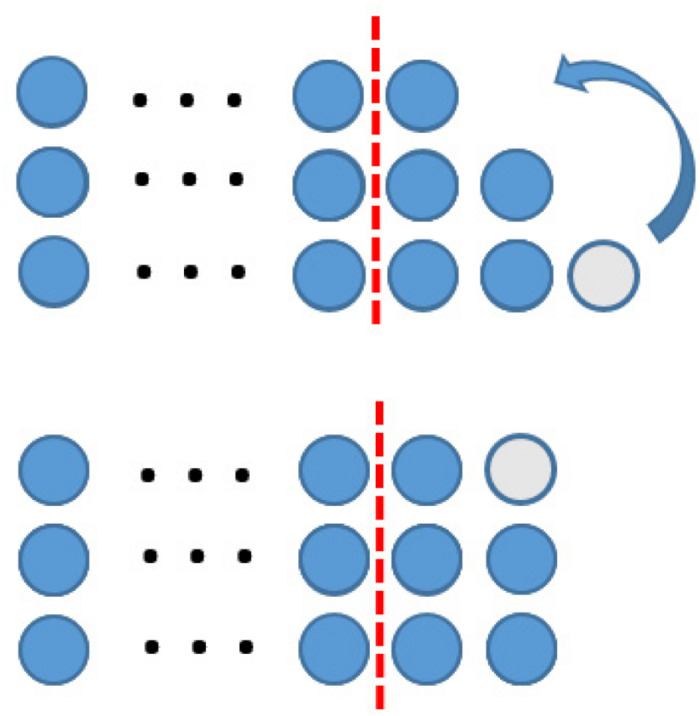
A proof using geometric variable (Figure similar to Kempen and Biehler, 2019a, p. 736).

In the context of figurate numbers, a special kind of symbol has been introduced to represent an arbitrary number of dots to express some kind of generality, too. Kempen and Biehler (2019a, p. 735) call this a “geometric-variable.” Geometric-variables allow the construction of mathematical proof in the context of figurate numbers expressing generality by its use of symbols (see Figure 8).

There are a lot of proofs collected in the literature making use of such geometric representations, called “charming proofs” (Alsina and Nelsen, 2010) or “proofs without words” (Nelsen, 1993, 2000; Alsina and Nelsen, 2009). From a meta point of view, such proofs are said to bear a special kind of explanatory quality (e.g., Hanna, 1990, 2018; Hemmi, 2006).

#### A Closer Look at the Idea of Explanatory Proofs

The famous distinction between proofs that prove and proofs that explain has been given by Hanna (1990). However, the idea of explanation allows for different approaches (Hanna, 2018). In the philosophy of mathematics, the explanatory quality is stressed by the connections between mathematical statements and their mutual relationships. From a pedagogical point of view, the idea of explanation is combined with some insights as to why a statement is true. In the following, we will refer to this pedagogical concept of explanation. We also refer to the characterization of proofs that explain, considered in Lockwood et al. (2019), for their description links the concept with the features of different representations systems that will be useful in the theoretical consideration from a semiotic point of view (see below). “We interpret, then, that a proof that explains allows for a prover to make meaning of whatever formal representation system he or she may be working with in order to connect ideas to some semantic system.” (ibid., p. 777). The idea of a semantic system is taken from the distinction between semantic and syntactic proof production that seems to be helpful for our discussion.

Weber and Alcock (2004) describe two different ways of producing mathematical proofs. The syntactical proof production is done by “manipulating correctly stated definitions and other relevant facts in a logically permissible way. […] The prover does not make use of diagrams or other intuitive and non-formal representations of mathematical concepts.” (ibid., p. 210). In a semantic proof production, a person uses instantiations of the mathematical objects to guide the formal inferences in the proving process. With instantiations, the authors describe “a systematically repeatable way that an individual thinks about a mathematical object, which is internally meaningful to that individual” (Weber and Alcock, 2004, p. 210). As Weber (2010b, p. 34) puts it, an explanatory proof “allows the reader to translate the formal argument that he or she is reading to a less formal argument in a separate semantic representation system”. (The author uses the term semantic representational system in opposite to a formal representational system). They give the following descriptions of an even function as an example: in a formal representational system, an even function satisfies the condition ∀*x* ∈ *ℝ*:*f*(*x*) = *f*(−*x*). In a semantic representational system, this concept might be described “informally as a function whose graph is symmetric around the *y*-axis” (ibid., p. 34).

**FIGURE 9:**
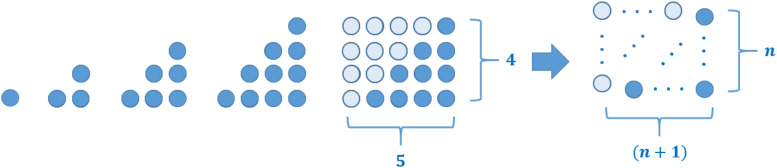
An explanatory proof for the sum of the first *n* ∈ *ℕ* natural numbers making use of figurate numbers.

This conceptualization of proof that explains gives a hint of why such explanatory proofs often make use of geometric descriptions to reach the conclusion: The representation system of figurate numbers can be considered to be such a semantic representation system, as it constitutes a non-formal way for communicating mathematical ideas. In this sense, proofs making use of geometric representations are considered to have a special kind of explanatory quality. As an example, we give an explanatory proof for the formula $1+2+\ldots +n=\frac{n\,\left(n+1\right)}{2}$ (see Figure 9).

### Figurate Numbers for Educational Purposes

In elementary school, figurate numbers can be used to get insights into the nature of natural numbers and the decimal systems and to promote mathematics as a science of patterns (e.g., Steinweg, 2002). Even at elementary school, figurate numbers offer the possibility to discuss generality and to introduce students to the idea of reasoning (e.g., Söbbeke and Welsing, 2017). Sequences of figurate numbers can be used in middle school to foster algebraic thinking (e.g., Rossi Becker and Rivera, 2006; Britt and Irwin, 2008). Moreover, the context of figurate numbers may serve as a playground to perform exploration and conjecturing in the interplay of algebra, arithmetic, and geometry (e.g., Weaver, 1974; Flores, 2002). For the context of first-year pre-service teachers at university, Kempen (2019, p. 21) highlights several benefits of the use of figurate numbers. Their use…

• … offers the possibility to take up students’ prior experiences from school mathematics, also concerning mathematical reasoning,
• … offers a non-symbolic language for the pre-service teachers they can use in their daily life as a teacher in the future,
• … makes it possible to involve the students in conjecturing and proving and helps to highlight the process aspect of mathematics,
• … may help to highlight the advantages of the mathematical symbolic language in comparison.

Moreover, in the comparison of the single test of some concrete examples, generic proofs and so-called ‘formal proofs’ making use of algebraic variables, the important distinction between purely empirical verification and the matter of generality in mathematical proofs can be stressed.

## Theoretical Considerations

### Diagrammatic Reasoning

In this section, we will analyze the use of figurate numbers from a semiotic point of view. This will help us discuss possible obstacles in their usage when reading and constructing mathematical proofs.

Peirce (1839–1914) uses the word “diagram” in a wide sense. He calls those signs and their combinations “diagrams” that can be used, read and understood in the context of a wider representational system, where the rules for dealing with the diagrams are constituted. As an example, we mention the following diagrams: “*a*^2^,” “2*x* + 5*y* + 6*x*,” and “7*b*^2^.” The meaning of the signs, their combinations and the possibility of transformations are given by the representational system of algebra. From a semiotic point of view, the area of figurate numbers can be considered to be such a representation system, too. Fischer (2010) describes the corresponding rules for dealing with the symbols:

Natural numbers are represented by the quantity of dots. Summation corresponds to joining amounts of dots, multiplication to the duplication of dots. Subtraction is done by eliminating dots (by erasing or crossing them). Dividing means to divide the dots into equal subgroups. (ibid., p. 86; our translation).

Following Peirce’s semiotic theory and his view on mathematics as “diagrammatic reasoning” (Hoffmann, 2003; Dörfler, 2008), the work in a representational system presupposes certain knowledge (“collateral knowledge”) about this system (Hoffmann, 2005). This knowledge comprises facts about the construction of diagrams, their usage and the interpretations of possible results. In some sense, this collateral knowledge can be seen as an implicit instruction manual for the use of the whole representational system. When performing or learning mathematics, one has to have the corresponding collateral knowledge, in order to work with the diagrams used or offered.

Peirce describes the mathematical activity making use of diagrams as the essential feature of mathematics:

By diagrammatic reasoning, I mean reasoning which constructs a diagram according to a percept expressed in general terms, performs experiments upon this diagram, notes their results, assures itself that similar experiments performed upon any diagram constructed according to the same percept would have the same results, and expresses this in general terms (Conference on Sensation-Mediation-Perception, 2012, p. 2).

This sequence of four phases [(i) construction of a diagram, (ii) performing experiments, (iii) observing the results, and (iv) determining the overall generality] describes the way new insights are gained in mathematics (see Dörfler, 2006, p. 211). The idea of diagrammatic reasoning, considered as the basic activity in mathematics, can be transmitted to the concept of mathematical proof. We will illustrate this by stating two different proofs concerning the claim “The sum of an odd number and its double is always odd.”

The proving process for a so-called “formal proof” is shown in Table 1.

**TABLE 1:** Description of the proving process for a so-called **“**formal proof**”** following the concept of diagrammatic reasoning.

Phase 1: Construction of the diagram	One possibility to prove this claim is by constructing diagrams in the representational system of algebra. Here, one needs an odd number, its double and the sum.	2*n* + 1,2⋅(2*n* + 1) with *n* ∈ *ℕ*_0_, 2*n* + 1 + 2⋅(2*n* + 1)
Phase 2: Performing experiments	The constructed diagrams can be transformed according to the rules of the representational system. (This transformation can be done either in an exploratory manner or with a certain objective).	= 3⋅(2*n* + 1) = 6*n* + 3 = 2⋅(3*n* + 1) + 1
Phase 3: Observing the results	The constellation of diagrams obtained can be read and interpreted according to the rules of the representation system. Here, the result can be understood as an odd number.	2(3*n* + 1) + 1 is an odd number, because (3*n* + 1) ∈ *ℕ*.
Phase 4: Determining the overall generality	The correctness of the result in a syntactical sense is a consequence of the correct usage of operations and transformations and the consistency of the representational system. The final insight follows by the interpretation of the final diagram and the link with the initial conjecture.	Accordingly, the sum of an odd number and its double is always odd.

Overall, the meaning of the collateral knowledge in the different phases as well as for the whole proving process becomes clear. We will compare this use of diagrams and the meaning of the corresponding collateral knowledge when dealing with the representational system of figurate numbers in the context of mathematical proof.

The corresponding proof in the representational system of figurate numbers (using geometric variables) is shown in Table 2.

**TABLE 2:** Description of the proving process for making use of figurate numbers following the concept of diagrammatic reasoning.

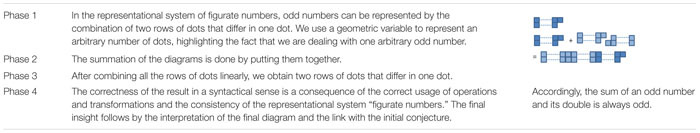

At this stage, we would like to highlight that several representational systems can be used to perform mathematical proving. The quality of a representational system has to be judged in comparison to its usefulness in this context. On the one hand, the writer of the proof has to have the corresponding collateral knowledge to construct such proofs. On the other hand, the reader of the proof also has to have this knowledge, to be able to read and to understand the proof correctly. Learners have to acquire certain collateral knowledge before they can be successful in working with any (geometric) representation.

### Some Insights From Cognitive Psychology

In this section, we will enrich the discussion about the use of figurate numbers by referring to different strands from cognitive psychology. After discussing basic aspects of understanding and the schema theory (see section “Diagrammatic Reasoning”), we will have a look at the perception of figurate numbers from the area of Gestalt psychology (see section “Some Insights From Cognitive Psychology”). Finally, the specific role of pictures and texts for understanding are revisited.

#### Understanding and the Extension of Existing Schema

From the perspective of cognitive psychology, the meaning of previous knowledge for learning is highlighted. Understanding is conceptualized as the integration of new information into the existing knowledge to build new schema (see Lee and Seel, 2012 for a summarized description). When working with (geometric) representations, this previous knowledge concerns semantical and syntactical issues. Since one person’s knowledge has an individual character, the process of understanding is an individual matter, too. However, the process of understanding (of getting new insights) must not be considered to be just some kind of accumulation. New information is integrated into one person’s existing knowledge and leads to elaboration, to the extension of existing schemata (Axelrod, 1973; Minsky, 1975; Collins et al., 1980). Following the perspective of the schema theory, one’s knowledge is organized and arranged in a specific way. DiMaggio (1997) brings in the schema aspect here: a schema describes a pattern of thought that organizes categories of information and the relationships between them. In this sense, the knowledge about the use and meaning of (geometric) representations is organized as a whole and constitutes a so-called schema. Combined with the concept of proof, the corresponding schema becomes evident. Again, a learner has to be acquainted with an adequate schema before being able to work with such representations or gain new insights from their usage.

When being confronted with a mathematical claim, a learner might activate the schema ‘figurate numbers’ in the context of proving. Activating this schema, several ‘blank spaces’ arise that have to be filled with respective knowledge:

– Geometrical representation (start): what kind of geometrical representation (e.g., shape) seems to be appropriate to represent the situation given in the context of the claim?
– Operations (start): which operations in the context of figurate numbers seem to be appropriate for being a translation of the operations mentioned in the given claim?
– Transformations: which transformations in the context of figurate numbers can be used to verify the given claim?
– Geometrical representation (end): what kind of geometrical representation should be reached after the transformations were done? What geometrical arrangement is considered to be a translation of the desired mathematical results?
– On a meta level: (1) why and when to use figurate numbers? (2) why is it possible and legitimate to perform mathematical proving with figurate numbers?

It becomes obvious that these demands have to be handled on top of the mathematical problem itself. This is also the case when using the algebraic language, but normally, learners have much more experience in using the algebraic language and therefore have a more complete schema in this case.

#### Some Remarks on Perception of Arrangements From the ‘Gestalt Psychology’

When dealing with figurate numbers, the question arises: why and how do we perceive such elements as arrangements in larger structures? For using figurate numbers to do mathematics and/or to grasp a general idea in a given pattern, it might be necessary to realize different structures within the whole. We take Figure 2 as an example: in this Figure, a 4 times 4 square is given. The transition from the previous to the given one results from seeing the following elements: the previous square (3 times 3), the two newly placed sides at the top and the right and the new point at the top right corner. Finally, for a general understanding of the transition from one square (number) to the next, this concrete pattern has to be recognized as a general one. The coming together of these aspects are necessary for obtaining the intended insight. This requires seeing one pattern in different ways. The Gestalt psychology gives some hints to why this activity might be problematic. Wertheimer (1938, p. 71; emphasis in original) describes this phenomenon as follows:

The concrete division which I *see* is not determined by some arbitrary mode of organization lying solely within my own pleasure; instead I see the arrangement and division which is given there before me. And what a remarkable process it is when some other mode of apprehension *does* succeed!

This author names several principles trying to explain the arrangement of stimuli perceived. Such impressions rely among others on the factor of proximity (this concerns the distance between individual elements) and the factor of similarity (the tendency to band similar or equal elements together)^1^. We cite two short examples (ibid., p. 72 and 74) to illustrate these principles.

**FIGURE 10:**
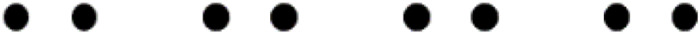
Sequence to illustrate the “factor of proximity” (patterns similar to Wertheimer, 1938, p. 72).

**FIGURE 11:**
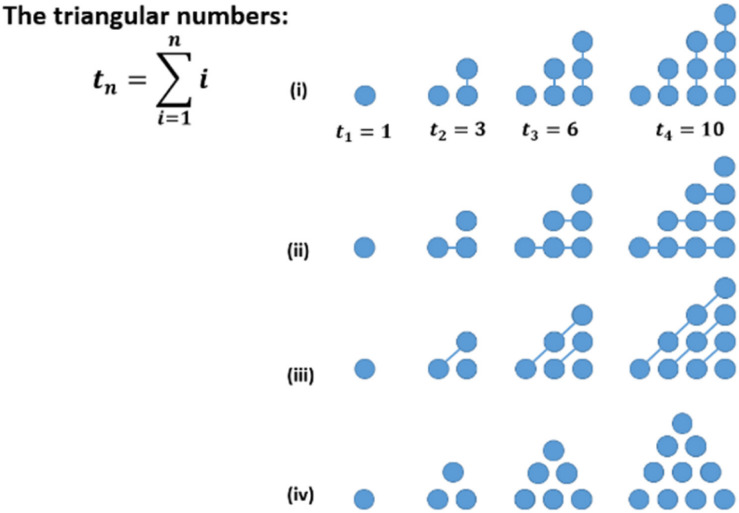
Different geometric interpretations of the triangular numbers.

Having a look at the sequence shown in Figure 10, one tends to ‘see’ naturally groups of two dots being near to each other (so to say the sequence “*ab | cd | ef | gh*”). Somehow it would be possible to always group the two dots next to the gap (“*a | bc | de | fg | h*”), which tends to be much harder. As Wertheimer puts it: (ibid., p. 73; emphasis in original): “[…] that form of grouping is most natural which involves the smallest interval. They all show, that is to say, the predominant influence of what we may call *The Factor of Proximity*.” In the sequence shown in Figure 11, all distances between the dots are exactly the same. However, the picture seems to contain vertical rows of dots. One might try to see a pattern of horizontal lines, but this tends to be harder. Accordingly, the factor of similarity describes the tendency to group similar elements.

**FIGURE 12:**
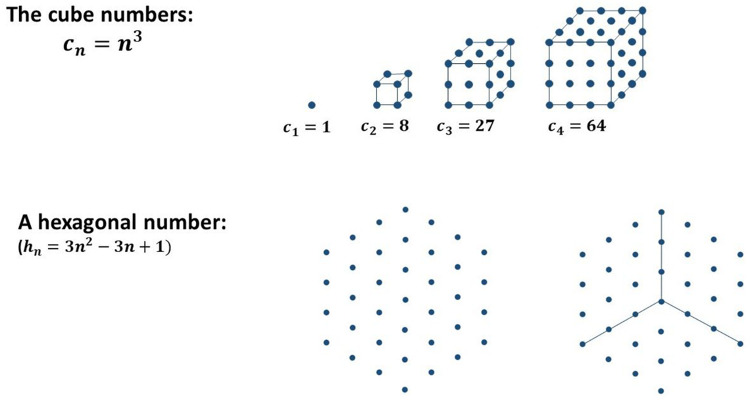
The connection between cube numbers and hexagonal numbers.

By making use of such principles (implicitly), one’s perception of figurate numbers (e.g., their geometrical shape as a whole or the phenomenon of detecting several sub-groups in the whole figure) might be explained.^2^ A first and simple example is given in Figure 12. The way one interprets the pattern of the triangular numbers has effects on the geometrical interpretation of the corresponding formula. Following interpretation (i), the new number line is always added on the right-hand side. Accordingly, the previous number is detected on the left in the actual pattern. In interpretation (ii), the new number line is added below the former pattern and (iii) offers a diagonal interpretation. Finally, the representation as a pyramid (iv) offers some more interpretation. However, one interpretation is necessary for a person for ‘seeing’ the connection between the given formula and the corresponding geometric shapes. It becomes obvious, that different interpretations can lead to a number of misunderstandings between teachers and learners or among the learners.

**FIGURE 13:**
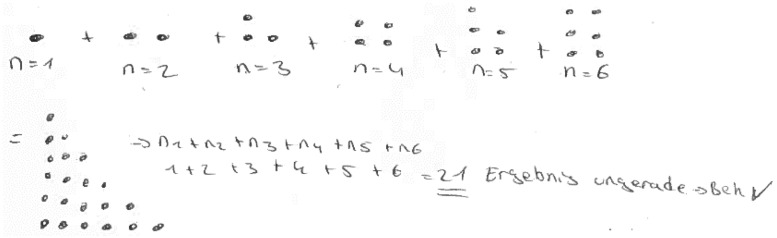
A student answer, which belongs to the category “empirical” [“=21 result odd → claim”].

Another example might be the fact, that the sum of two consecutive triangular numbers is always a square number. A more complex example of seeing a subpattern in the whole is given in Figure 13. Do you ‘see’ why the difference of two cube numbers is always a hexagonal number (Figure 13)? Adding three lines makes it much easier to see this relationship. The respective connection in the transition from one cube to the next can be calculated easily: *c*_*n*_−*c*_*n*−1_ = *n*^3^−(*n*−1)3 = *n*^3^−[*n*^3^−3*n*^2^ + 3*n*−1] = 3*n*^2^−3*n* + 1 = *h*_*n*_.

In accordance to the patterns or shapes perceived, operations on these figures (see section “Diagrammatic Reasoning”) might be considered as “pro-structural,” when being in line with the perceived structures or when leading to new ones, or as “contra-structural” (ibid., p. 76), when destroying some structure. Thus, the perception of a shape might guide one’s operations.

However, seeing a different arrangement after perceiving the first one tends to be difficult (Wertheimer, 1938, p. 71):

[…] one sees a series of discontinuous dots upon a homogeneous ground not as a sum of dots, but as figures. Even though there may here be a greater latitude of possible arrangements, the dots usually combine in some “spontaneous,” “natural” articulation – and any other arrangement, even if it can be achieved, is artificial and difficult to maintain.

For each individual, through the coming together of the various named principles and the individual’s experiences, a certain initial interpretation of what has been experienced emerges. If one tries to see another interpretation, the earlier stimuli must be overcome.

From a meta-level, it seems to be significant, not only for the phenomenon of figurate numbers, that some kind of quantity is translated into orderly spaced identical elements. In our cases, these orderly spaced identical elements are considered to convey special kinds of insights. However, this principle can be detected in other parts of mathematics, too. As an example, we point to the meaning of such representations for estimating quantities (e.g., Hansen et al., 2015). Another example is the translation of quantities into position in space as one basic principle of many data graphs such as scatter plots (some nice examples are discussed in Garcia-Retamero and Cokely, 2017).

#### About the Role of Figurate Numbers Seen as ‘Pictures’

Our focus is on the interplay of mathematical content and the use of figurate numbers. To be successful in achieving understanding, learners have to combine the given content with its interpretation in the context of figurate numbers and to integrate this information into one coherent mental representation. Since figurate numbers are a specific type of representation that somehow resembles a picture, it could be assumed that, unlike conventional texts, they could fulfill different functions in the process of understanding. Accordingly, we will take a first look at the role that ‘pictures’ play in our everyday process of understanding. [For this discussion, we will shortly leave the interpretation of pictures and its parts as diagrams in a wider representational system in the sense of Peirce (see above), for our intention is to highlight a normal or naïve role of pictures for the individual in the context of understanding].

As a part of our living in the real world, we seem to have learned that reading a text is about acquiring information and about constructing mental models. Pictures, particularly given in addition to a text, are about reading off information (compare Zhao et al., 2020). Furthermore, pictures can constrain the interpretation of a text (Ainsworth, 1999) and serve a scaffolding function for constructing mental representation (e.g., Eitel and Scheiter, 2015) that might lead to the construction of deeper understanding (Ainsworth, 2008). However, research has shown, that learners often fail to exploit such advantages and the use of (several) representation might hinder learning (ibid.). When one works with figurate numbers, the ‘pictures’ themselves become the center of interest. At first glance, it might seem quite unnatural that these pictures should contain all relevant information (and not a given text), this phenomenon might contradict previous experiences. In addition, one is also asked to work with these pictures; the diagrams should be (intentionally) changed and new information should be taken from the result. This change in function may prevent learners from fully exploiting the potential of the figurate numbers.

For the process of problem solving, the search for a suitable representation of the problem can serve as a promising heuristic (see section “Geometric Representations in the Context of Problem Solving”). A type of representation will emphasize certain or characteristic features of the initial problem (see also Dunbar, 1998, p. 294). In this sense, a change of presentation will also change the problem: This change may affect the initial state, the target state and/or the set of applicable operations (ibid.; see also section “Diagrammatic Reasoning”). In summary, it can be said that a problem discussed in another representation system can be considered a different problem.

## Findings From Our Research Underlining the Theoretical Considerations Above

In this section, we will recapitulate findings and experiences from our empirical research in the context of figurate numbers. The research presented here touches upon the following aspects: students’ proof construction making use of figurate numbers (see section “Students’ Proof Construction With Making Use of Figurate Numbers”), students’ perceived explanatory power, conviction, and proof-acceptance (see section “Proof-Acceptance, Explanatory Power, and Conviction”), and students’ perception of proofs making use of figurate numbers (see section “Students’ perception of proofs making use of figurate numbers”). Due to the size of this paper, we will only report on the main findings. For deeper descriptions of the methodology used and further results the relevant references will be given.

### Students’ Proof Construction With Making Use of Figurate Numbers

The authors investigated pre-service teachers’ proof construction in the winter term 2013/2014 (Biehler and Kempen, 2015) and 2014/2015 (Kempen, 2017, 2019) in the context of the transition-to-proof course “Introduction into the culture of mathematics.” In both years, the students were asked to prove a given claim in the final exam of the course by using four different kinds of proofs they had learned about before. The claim to be proven was: “The sum of six consecutive natural numbers is always odd.” These four different kinds of proofs comprise:

(1) One proof with concrete examples making use of natural numbers. In this case, the overall argument to verify the given claim in general was explicated in a narrative (“generic proof with numbers”^3^).
(2) One proof with concrete examples making use of figurate numbers. In this case, the overall argument to verify the given claim in general was explicated in a narrative (“generic proof with figurate numbers”).
(3) One proof making use of figurate numbers and geometric variables to highlight the general quality of the argument given in the geometrical representation (“proof with figurate numbers making use of geometric variables”).
(4) One so-called formal proof making use of algebraic variables (“formal proof”).

The authors developed a set of categories for summarizing all proving attempts and for comparing the results evenly between the different kinds of proof. A summarized version of the set of categories is described below (compare Kempen, 2017, p. 388 f.); the examples illustrating the categories concern the generic proof with figurate numbers. The claim to be proven is mentioned above.

**FIGURE 14:**
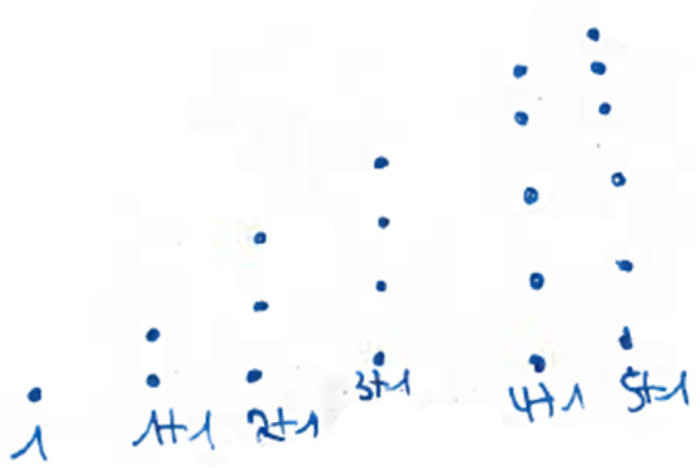
A student answer, which belongs to the category “pseudo.”

**FIGURE 15:**
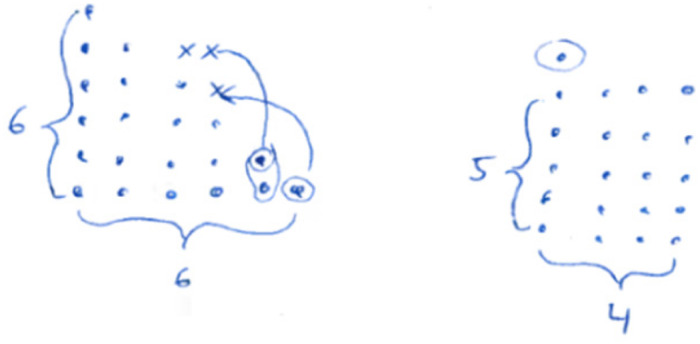
A student answer, which belongs to the category “fragmentary.”

**FIGURE 16:**
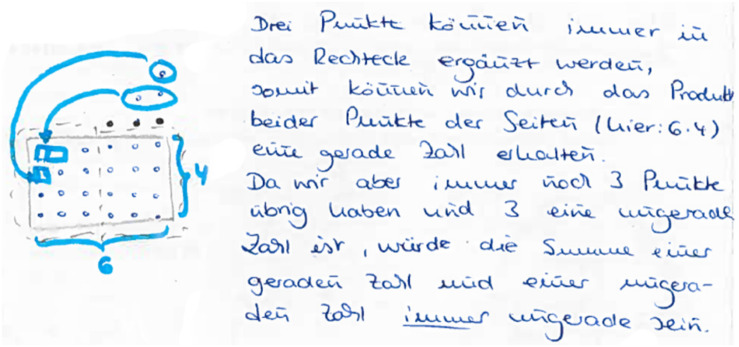
A student answer, which belongs to the category “sound argument” [“Three points can always be used for completing the rectangle. Thus, we obtain the product by multiplying the sides (here: 6⋅4)” which is even. Since there are always three points left, and three is an odd number, the sum of the even and the odd number will always be odd].

**FIGURE 17:**
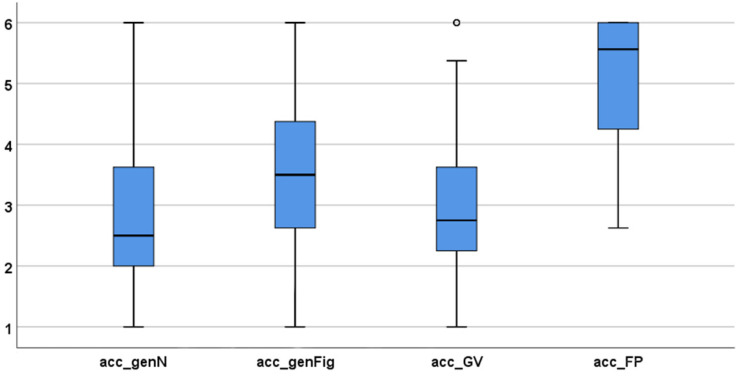
Boxplots concerning students’ measured proof acceptance.

(1) ** n. p.:** not processed.
(2) **Empirical:** The truth of the statement is inferred from a subset of (concrete) examples (see Figure 14).
(3) **Pseudo:** the answer is given by merely stating or paraphrasing the statement that the sum is always odd/wrong solutions/irrelevant information/construction (see Figure 15).
(4) **Fragmentary:** only fragmentary information is given/meaningful arrangement of figurate numbers without further information (see Figure 16).
(5) **Sound argument:** the students derives the conclusion from a connected argument and from generally agreed facts of principles that might contain (minor) inaccuracies (see Figure 17).

In both years, the students were asked to prove the given claim with all four kinds of proof in the final exam of the course. The corresponding results are shown in Table 3.

**TABLE 3:** Results [%] concerning students’ proof productions in the context of the course “Introduction into the culture of mathematics” concerning four different kinds of proofs (“genN,” generic proof with numbers; “genFig,” generic proof with figurate numbers; “GV,” proof with geometric variables; “FP,” formal proof).

	**Winter term 2013/14 (*n* = 139)**				**Winter term 2014/15 (*n* = 107)**			
		
	**GenN**	**FP**	**GenFig**	**GV**	**GenN**	**FP**	**GenFig**	**GV**
n. p.	3	3	6	18	0	2	1	4
Empirical	7	0	7	0	1	0	0	0
Pseudo	22	15	37	45	6	8	14	34
Fragmentary	14	3	24	9	11	6	36	10
Sound argument	54	79	27	28	82	84	50	52
Sum	100	100	100	100	100	100	100	100

First, we would like to stress that several students did not even try to solve the given task by using figurate numbers (“GenFig” + “GV”) in the winter term 2013/2014, even though, they were sitting an exam to pass the course. Moreover, the higher percentage of pseudo answers and the lower results of proving attempts belonging to the category “sound argument” when working with figurate numbers (“GenFig” + “GV”) are astonishing in both years.^4^

To sum up, these results highlight students’ difficulties in making use of figurate numbers to construct mathematical proofs.

### Proof-Acceptance, Explanatory Power, and Conviction

Kempen and Biehler (2019b) investigated the perceived explanatory power, conviction, and proof acceptance concerning the four different kinds of proof (see above) in the context of pre-service teachers at the University of Paderborn in Germany.

We will rely on the following research questions taken from the study of Kempen and Biehler (2019b)

(i) How do pre-service teachers rate the different kinds of proofs concerning the perceived explanatory-quality and conviction at the beginning of their university studies?
(ii) How can students’ proof acceptance of the four kinds of proofs be described?

While Kempen and Biehler (2019b) investigated the changes in students’ proof perception and acceptance while attaining the course “Introduction into the culture of mathematics,” we will have a close look at the corresponding results from the pre-test at the beginning of the course.

To answer the research question (i), the students were asked to rate one proof of each kind (see above) concerning the perceived explanatory power and conviction on a six-level Likert scale ([1] “totally Disagree” … [6] “totally agree,” see Table 4). We cite the example for the so-called formal proof (Kempen and Biehler, 2019b, p. 39 f.) for the claim “For all natural numbers *a*,*b*,*c*: If *b* is a multiple of *a* and *c* is a multiple of *a*, then (*b+c*) is a multiple of *a*.”

**TABLE 4:** The items concerning “conviction” and “explanatory power” for the rating of the four different kinds of proof.

**The reasoning…**	**Totally disagree**			**Totally agree**		
	**[1]**	**[2]**	**[3]**	**[4]**	**[5]**	**[6]**
Convinces me that the statement holds in every case.						
Explains why the statement is true.						
…

Let *a*,*b*,*c* be natural numbers. Since *b* is a multiple of *a*, there exists a natural number *n* with: *n*⋅*a=b*. Since *c* is a multiple of *a*, there exists a natural number *m* with: *m*⋅*a=c*. We have: *b* + *c* = *n*⋅*a* + *m*⋅*a* = *a*⋅(*n+m*). Since (*n+m*) is a natural number, (*b+c*) is a multiple of *a*. Q.e.d.

As indicated in Table 4, students were asked to rate other statements concerning the given proofs, too. These statements comprised the aspects verification, interpretation as purely empirical verification, the existence of counterexamples, the importance of variables, the interpretation as testing of concrete cases and correctness. The mean of the different ratings for each kind of proof was considered to be one’s score in “proof acceptance.” I.e., a high scale value represents a high level of acceptance concerning a given ‘proof’ and vice versa. This construction of one scale was confirmed by a corresponding factor analysis. The reliabilities of the constructed scales for the four kinds of proof out of the eight items were very high (all Cronbach’s alpha > 0.88). Accordingly, we used the following conceptualization for “proof acceptance” to answer the research question (ii):

“‘proof acceptance’ is conceptualized as the extent to which an individual perceives verification, conviction and explanation when reading a mathematical proof combined with the extent, the reader does consider the reasoning to be a correct mathematical proof” (ibid., p. 31).

We quote the corresponding results:

With regard to both conviction and explanatory power, the formal proof achieved the highest ratings (Table 5), whereas the proof with geometric variables achieved the lowest. The results concerning the generic proofs are located between these kinds of proofs. All differences concerning the medians are pairwise highly statistically significant (*p* ≤ 0.001, Wilcoxon-test) with medium and high effect sizes (see Table 6).

**TABLE 5:** Statistical data concerning the items “conviction” and “explanatory power” (“genN,” generic proof with numbers; “genFig,” generic proof with figurate numbers; “GV,” proof with geometric variables; “FP,” formal proof).

	**Conviction**				**Explanatory power**			
	**genN**	**genFig**	**GV**	**FP**	**genN**	**genFig**	**GV**	**FP**
*n*	74	74	68	72	74	74	68	72
Mean	3.32	4.38	2.96	5.35	3.82	4.50	2.85	5.15
Median	3.00	5.00	3.00	6.00	4.00	5.00	3.00	6.00
*SD*	1.664	1.411	1.688	1.050	1.511	1.274	1.730	1.206
Minx	1	1	1	2	1	1	1	2
Max	6	6	6	6	6	6	6	6

**TABLE 6:** Statistical significance of the differences between the medians concerning “conviction” and “explanatory power” (*p*-value, Wilcoxon-test) with effect sizes [Pearson’s correlation coefficient (r)].

	**Conviction**			**Explanatory power**		
	**genFig**	**GV**	**FP**	**genFig**	**GV**	**FP**
genN	*p* < 0.001 (*r* = 0.47)	–	*p* < 0.001 (*r* = 0.71)	*P* = 0.001 (*r* = 0.39)	*p* = 0.001 (*r* = 0.41)	*p* < 0.001 (*r* = 0.58)
genFig	–	*p* < 0.001 (*r* = 0.59)	*P* < 0.001 (*r* = 0.49)	–	*p* < 0.001 (*r* = 0.63)	*P* = 0.001 (*r* = 0.40)
GV	–	–	*p* < 0.001 (*r* = 0.75)	–	–	*p* < 0.001 (*r* = 0.70)

The results concerning students’ proof acceptance are shown in Table 7 and Figure 17. The score concerning the generic proof with numbers (mean of 2.79) was quite low, as was the acceptance of the proof with geometric variables. Again, the formal proof achieved the highest score (mean: 5.15).

**TABLE 7:** Statistical data concerning proof acceptance scales.

	**acc_genN**	**acc_genFig**	**acc_GV**	**acc_FP**
*n*	74	74	67	72
Mean	2.79	3.67	2.96	5.15
Median	2.50	3.50	2.88	5.63
*SD*	1.18	1.27	1.27	1.02
Min	1.00	1.00	1.00	1.00
Max	6.00	6.00	6.00	6.00
Cronbach’s alpha	0.886	0.912	0.896	0.939

All differences between the means are highly statistically significant (*p* ≤ 0.001; *t*-test) with medium to high effect sizes, except for the difference between the generic proof with numbers and the proof with geometric variables (see Table 8).

**TABLE 8:** Statistical significance of the differences between the means of the acceptance scores (*p*-value, *t*-test) with effect sizes (Cohen’s *d*).

	**acc_genFig**	**acc_GV**	**acc_FP**
acc_genN	*p* < 0.001 (Cohen’s *d* = 0.663)	*p* = 0.412 (–)	*p* < 0.001 (Cohen’s *d* = 2.229)
acc_genFig	–	*P* = 0.001 (Cohen’s *d* = 0.427)	*p* < 0.001 (Cohen’s *d* = 1.269)
acc_GV	–	–	*p* < 0.001 (Cohen’s *d* = 1.845)

To sum up, the students in our study struggled with the interpretation of figurate numbers in the context of proving. The use of these geometric representations in such proofs did not lead to an increased perception of conviction or explanatory power. On the contrary, the proof making use of algebraic variables (the ‘formal proof’) was perceived as the most convincing and explanatory argument. The same is true for the measured proof-acceptance values^5^.

### Students’ Perception of Proofs Making Use of Figurate Numbers

Kempen and Biehler (2015) conducted an interview study with 12 first-year pre-service teachers to investigate students’ perceptions of proofs making use of concrete examples in elementary number theory. These students participated in the course “Introduction into the culture of mathematics,” where they were introduced to the concept of proving. In the context of the course, the varying use of concrete examples, figurate numbers, and algebraic variables played an important role (see Kempen and Biehler, 2019a, b). In this research study, the students were asked to work on the following task: “Prove or disprove: If one takes a natural number and adds its square, the result will always be divisible by 2.” After students’ initial answers, an interview phase followed. Here, the students were asked to explain their proving attempts to reason why they used the respective approach in contrast to the other ones they had learned in the course. We transcribed each session and analyzed the transcripts and students’ proof constructions. We looked for common and characteristic patterns in students’ comments to categorize them as cases of a certain type.

This study reveals some interesting results concerning learners’ perspective on the usage of figurate numbers. Following students’ responses in the interview, proofs making use of figurate numbers (i) are hard to construct because one always has to have a special “idea” and (ii) can be harder to understand than formal proofs. As an example, we cite the following statements from three different students (taken from Kempen, 2019, p. 260 f.; authors’ translation):

[…] compared to the one with figurate numbers, but since [with figurate numbers; L. K.] you always need an idea first, right? That’s why I like it worse, compared to the formal proof, because one always has to have an idea.

[…] we know at an early age if we multiply a number by two, that the result is logically divisible by 2. Here [in the case of figurate numbers; L. K.] one has to consider horizontal/vertical, odd number above/even number below. The feeling of looking at and understanding is easier here [in the case of the formal proof; L. K.]. Here one shows, no matter which natural number you take, multiplied by two will be logically divisible by 2.

I find that [the formal proof; L. K.] is most understandable for everyone. If someone else were to look at it, he or she would most likely understand it, instead of such proofs with figurate numbers, where one would have to think over and over again.

These results point to the fact, that the use of figurate numbers (even for university students) cannot be considered as being that easy. Argument (i) points to the problems that have already been raised in the context of schema theory: starting with a mathematical claim, one has to translate the given information to the representational system of figurate numbers. This means that a geometrical interpretation of the given facts has to be undertaken. The second argument highlights the fact that the use of figurate numbers must not be considered as being easier than the use of the algebraic symbolic language. As already mentioned in the context of Peirce’s semiotic theory, dealing with a representational system has to be learned and practiced. In this way, learners might acquire the respective collateral knowledge to work in this system.

## Summary, Conclusion, and Implications for Teaching and Research

It has been shown above that figurate numbers can be used in mathematics in various ways, e.g., for illustrating, clarifying, and illuminating mathematical issues. Moreover, in the context of problem solving and proving, a change to this special kind of representational system and working with it can be considered to be a useful heuristic. Especially in the context of mathematical proof, working with such ‘semantic’ representational system (Lockwood et al., 2019) is said to increase the explanatory power of mathematical proof leading to so-called proofs that explain. Besides, the use of such representation is said to ease the transition to algebra and to contribute to a meaningful concept of variable. Finally, working within this field can constitute a playground for exploration, conjecturing, and proving in the interplay of algebra, arithmetic, and geometry.

However, the discussion of part of Peirce’s semiotic theory led to a closer look at the representational system ‘figurate numbers.’ For working with the corresponding symbols and signs, a special kind of knowledge (“collateral knowledge”) is necessary. This knowledge comprises facts about the construction of diagrams, their usage and the interpretations of possible results. Working with figurate numbers in mathematics (especially in mathematical proving) can be conceptualized as diagrammatic reasoning, i.e., reasoning by making use of such diagrams. It became clear that performing mathematics with figurate numbers or understanding someone else’s performance presupposes the existence of the corresponding collateral knowledge.

The discussion about necessary prior knowledge and the acquisition of new understanding could be elaborated by referring to cognitive psychology. Here, learning and understanding are combined with the integration of new information into the existing knowledge to build new schema. In addition to parts of knowledge referring to the use of such representations (an appropriate ‘translation’ of a mathematical issue to the system of figurate numbers, the choice of operations to achieve a selected aim) some meta-knowledge about the usage of such representations (e.g., “why and when to use them”) is necessary, too. Since geometric representations like figurate numbers fulfill distinct functions in the context of understanding and the construction of mental models, the question arose, as to how learning processes change while changing the representational system. Finally, it became obvious, that one problem or task changes fundamentally when changing the representational system, because the initial state of the problem, the goal state and/or the set of operations that can be applied will differ fundamentally. Besides, the semiotic considerations above hint toward the fact, that while changing a representational system, another collateral knowledge is necessary, that can be developed more or less than the previous one for each person. This is also true for the interpretation of learning and understanding by referring to a corresponding schema.

Insights from the Gestalt psychology made it possible to investigate the phenomenon of ‘seeing’ patterns within the arrangements of figurate numbers. However, corresponding principles of perception do not constitute universally valid rules, the individual experiences play another constitutive part. That is why the individual’s perception of geometric arrangements may be different to someone else’s. (The corresponding reading and understanding of a perceived geometric shape is again a matter of collateral knowledge). Working with figurate numbers demands a flexible perception about recognizing patterns, imaging future constellations, and eventually grasping a general idea. Furthermore, the identification of patterns does also affect the perception and awareness of possible operations or transformations that can be used, being “pro-structural” and “contra-structural.” Accordingly, the individual’s perception of a given arrangement may influence its choice of operations or transformations which, of course, also indicates the possibilities of achieving the respective goals and possible insights. The coming together of all these aspects illustrates the demands placed on learners when working with figurate numbers. Finally, the way of working with these ‘pictures’ for performing operations, achieving results, and getting new insights may contradict previous experiences about the role of pictures and texts in the context of learning.

In chapter 4, we summarized some findings from our own empirical research concerning the use of figurate numbers in a variety of aspects concerning the topic “mathematical proof.” As could be observed in every study, the students struggled with the use and the understanding of figurate numbers. This was somehow in contrast to the descriptions in the literature, highlighting the benefits of the use of figurate numbers for educational purposes. Concerning students’ proof productions, the learners struggled the most in constructing mathematical proofs by using figurate numbers. However, the students succeeded much better in construction generic proofs with numbers (instead of figurate numbers) and formal proofs. In this sense, the use of the representational system of Algebra seemed to be much easier for them than the one of figurate numbers and lead to the construction of proofs at a higher level. Concerning perceived explanatory power and conviction, the formal proof making use of algebraic variables always got the highest ratings. In this sense, the pre-service teachers in our study did not perceive a special kind of explanatory power and conviction in the context of the representational system ‘figurate numbers.’ Moreover, such proofs achieved significantly less scores concerning the individual’s “proof acceptance” than the formal proof. Taken together, these students seemed to appreciate especially the mathematical symbolic language in the context of proving. Results from our interview study could partly explain the results obtained. Students mentioned the necessity for a special “idea” when working with figurate numbers. When working with natural numbers (generic proof with numbers) or algebraic variables (formal proof), the students did not mention such challenges.

Finally, we will combine the theoretical considerations and the empirical findings. In the studies presented, the representational system of algebra (making use of algebraic variables in the context of elementary arithmetic) led to the biggest success when being used by students. These results can be explained by the fact that this representational system is the most used and practiced one in school mathematics. Other representational systems (as figurate numbers) are used less. Accordingly, students did not have enough time to acquire the corresponding collateral knowledge and to practice its use. Working within a representational system can be described by the four phases of diagrammatic reasoning [(i) construction of a diagram, (ii) performing experiments, (iii) observing the results, and (iv) determining the overall generality]. In all of these phases, a special kind of knowledge is necessary to cope with the respective aspects of a representational system. The lack of collateral knowledge will prevent the construction of correct mathematical proofs.

The named hints from schema theory highlighted the aspects, learners have to be (implicitly) aware of when working with figurate numbers in mathematics, too. When trying to prove a given claim, all aspects named in the given statement have to be transmitted to the representational system of figured numbers. Then, the conclusion has to be faced, again interpreted in the context of figurate numbers. Finally, this goal has to be achieved using the possible operations in this representational system. Again, students’ problems when working with figurate numbers can be partly explained by making use of such aspects from the schema theory. However, the corresponding understanding and interpretations belong to the individual’s perception which also affects the identification and selection of suitable operations. Accordingly, the given problem changes by undertaking a change of the given representational system and it also changes due to the individual’s perception. These perceptions could be elaborated by pointing to the Gestalt psychology. In addition, there is not only the need for perceiving and constructing a first pattern, as the initial state of a given problem. There are multiple arrangements and possibilities the learner has to recognize. A change in perception is difficult to achieve. However, this is a necessary prerequisite for making targeted transformations.

Both theoretical perspectives mentioned above highlight the necessity of corresponding prior knowledge for being able to work with and to understand the representational system of figurate numbers. In this sense, the use of such representations is no guarantee to lead to special insights. The explanatory quality of such ‘pictures’ or ‘proofs’ has to be considered as an ‘offer’ and not as a ‘present.’ The understanding of a representation is an individual process (of elaboration) and relies on the individual’s previous knowledge and perception. All kinds of representations (or representational systems) constitute a learning content at the first level. Even so-called ‘explanatory’ representations have to be read and to be understood by a certain reader, who has to have the corresponding collateral knowledge (in the sense of Peirce, see above). Considering learning as an active process based on one’s prior knowledge highlights the subjective nature and the relativity of the understanding of given representations. Accordingly, such ‘explanatory proofs’ making use of geometrical representations are not self-evident nor self-explanatory (see also Jahnke, 1984); ‘explanatory proofs’ cannot be considered to be explanatory by themselves.

These considerations lead to several implications for teaching: As Dörfler (2006) points out, learners have to perform several activities to get used to a representational system, i.e., to acquaint the corresponding collateral knowledge. These activities comprise (i.a.): manipulating (performing calculations) with the diagrams, performing experiments on the diagrams to explore their characteristics, investigating the relationships between such diagrams, inventing new diagrams, etc. Some examples of such activities can be found in modern textbooks. Kempen and Biehler (2019a) proposed some learning environment for first-year pre-service teachers to cope with different representational systems in the context of mathematical proof.

The perspective of schema theory highlights the questions, what kind of knowledge concerning the representational system as a whole is necessary to construct a coherent schema for dealing with this system in mathematics. (This knowledge also touches upon some kind of meta knowledge concerning mathematics). Such question can partly be discussed from the perspective of diagrammatic reasoning (see above). However, the Gestalt psychology stresses the individual’s perception in this context. Besides, affective factors might also contribute to the individual’s perspective.

The theoretical considerations above also lead to implications for research. The use of geometric representation in mathematical activities (like problem solving or conjecturing and proving) has to be investigated at different stages and in different institutions. Here, the aspect of “acceptance” should be considered, too, constituting a basis for the individuals work and understanding. This is also true when considering different research areas of mathematics and different representations in the context of proving (e.g., Weber, 2010a). Finally, the phenomenon of getting some ‘insights’ demands further research. Haider and Rose (2006) have proposed a way for detecting ‘insights’ empirically. Besides, philosophical investigations seem to be promising for conceptualizing this unique moment of ‘understanding-why’ (e.g., Lawler, 2019).

## Data Availability Statement

The datasets generated for this study are available on request to the corresponding author.

## Author Contributions

All authors listed have made a substantial, direct and intellectual contribution to the work, and approved it for publication.

## Conflict of Interest

The authors declare that the research was conducted in the absence of any commercial or financial relationships that could be construed as a potential conflict of interest.

## Footnotes

1 Concerning the perception of arranged stimuli, Wertheimer (1938) also mentions the factor of continuity and the factor of closure.

2 Similar principles for the grouping of patterns (proximity, collinearity, and good-continuation) could be verified empirically by Kubovy and van den Berg (2006).

3 Concerning the concept of generic proofs see Section “Figurate Numbers and Mathematical Proof.”

4 The different results between the 2 years can be explained by referring to several changes made in the whole course (see Kempen, 2019 for description of the whole research project).

5 This study was originally conducted with a bigger sample size. The students dealt with in Kempen and Biehler (2019b) are those, that could be tracked from the pre- to the post-test. The whole sample contains 145 pre-service teachers. The corresponding results are discussed in Kempen (2018). However, also in the whole sample, the overall results are nearly the same: the formal proof achieves the highest ratings and acceptance-score.
